# Haemophilus parainfluenza-Associated Aortic Root Abscess and Septic Embolic Stroke Post-transcatheter Aortic Valve Replacement: A Case Study

**DOI:** 10.7759/cureus.61757

**Published:** 2024-06-05

**Authors:** Tirumala Nischal Jasty, Jaswanth R Jasti, Smit Shah, Jared Green

**Affiliations:** 1 Internal Medicine, Tower Health Medical Group, West Reading, USA; 2 Cardiology, Tower Health Medical Group, West Reading, USA; 3 Cardiology, Reading Hospital, Tower Health, West Reading, USA

**Keywords:** hacek, embolic stroke, transcatheter aortic valve replacement, echocardiography, aortic root abscess, haemophilus parainfluenza

## Abstract

We present a rare instance of aortic root abscess and septic embolic stroke due to* Haemophilus parainfluenza *in a 75-year-old male patient who had undergone a recent transcatheter aortic valve replacement. The patient initially presented with generalized weakness and altered mental status. Blood cultures grew *Haemophilus parainfluenza*. Brain imaging showed multiple infarcts with some areas of hemorrhagic conversion. Echocardiography demonstrated an aortic root abscess as well as vegetation on the tricuspid valve. Surgical correction was deemed high risk; hence, management involved intravenous (IV) antibiotics, resulting in the resolution of both symptoms and the abscess.

## Introduction

Transcatheter aortic valve replacement (TAVR) is a minimally invasive procedure increasingly used in the treatment of aortic stenosis, especially in high-risk surgical patients [1]. While the procedure has revolutionized the management of this condition, it is not without complications, one of them being infective endocarditis (IE) leading to systemic embolization [2]. *Haemophilus* species, part of the *Haemophilus*, *Aggregatibacter*, *Cardiobacterium hominis*, *Eikenella corrodens*, and *Kingella* species group (HACEK group), are normal inhabitants of the oropharynx. While these organisms are commonly benign, they are rare but notable causes of IE [3]. Here, we report a rare case of *Haemophilus parainfluenza* aortic root abscess and septic embolic stroke following a recent TAVR procedure. This case highlights the importance of timely diagnosis and the challenges in managing post-TAVR infective complications.

## Case presentation

**Figure 1:**
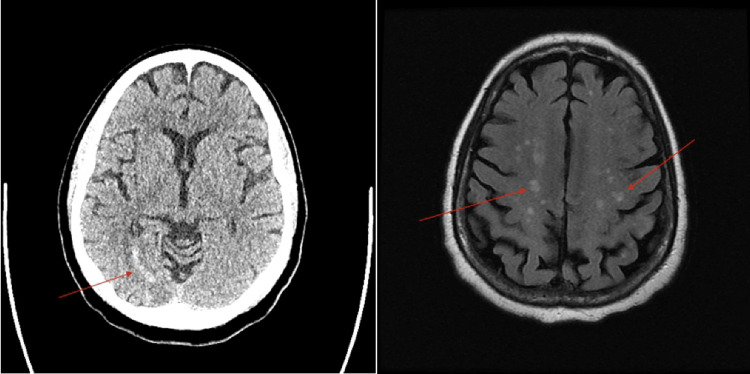
CT scan of the head in axial section (left) reveals an infarction in the right PCA area (red arrow). MRI of the brain in axial section (right) shows numerous infarcts, suggesting an embolic cause (red arrows). CT: Computed tomography; PCA: posterior cerebral artery; MRI: magnetic resonance imaging

A 75-year-old male with a history of coronary artery disease and aortic stenosis post-recent TAVR (two months ago) presented to our emergency department with generalized weakness and altered mental status. Four weeks earlier, he had an outpatient office visit when no clinical changes, electrocardiogram (EKG), or transthoracic echocardiogram (TTE) abnormalities were noted. At presentation, the patient was febrile with a temperature of 101.3°F (38.5°C) but hemodynamically stable on room air. With the patient being encephalopathic, history was limited; however, a review of past medical history was significant for other comorbidities, including chronic lymphocytic leukemia with anemia and thrombocytopenia, hypertension, and hyperlipidemia. The patient was only oriented toward self; physical examination was otherwise unremarkable. Complete blood count with differential showed a white blood cell count of 20,700 K/uL (baseline: 10000-11000 K/uL) with neutrophilic predominance, hemoglobin of 8 g/dL (at baseline), and a platelet count of 98,000 K/uL (at baseline). Serum chemistry was unremarkable. Lactic acid was elevated at 5.9 mmol/L (reference: 0.6-1.4 mmol/L). High-sensitivity troponin was within normal limits, and the EKG showed a normal sinus rhythm. A non-contrast computed tomography (CT) and magnetic resonance imaging (MRI) of the brain revealed multiple infarcts in the middle cerebral artery (MCA) and posterior cerebral artery (PCA) territories with hemorrhagic conversion, suggesting an embolic source (Figure 1).

**Figure 2:**
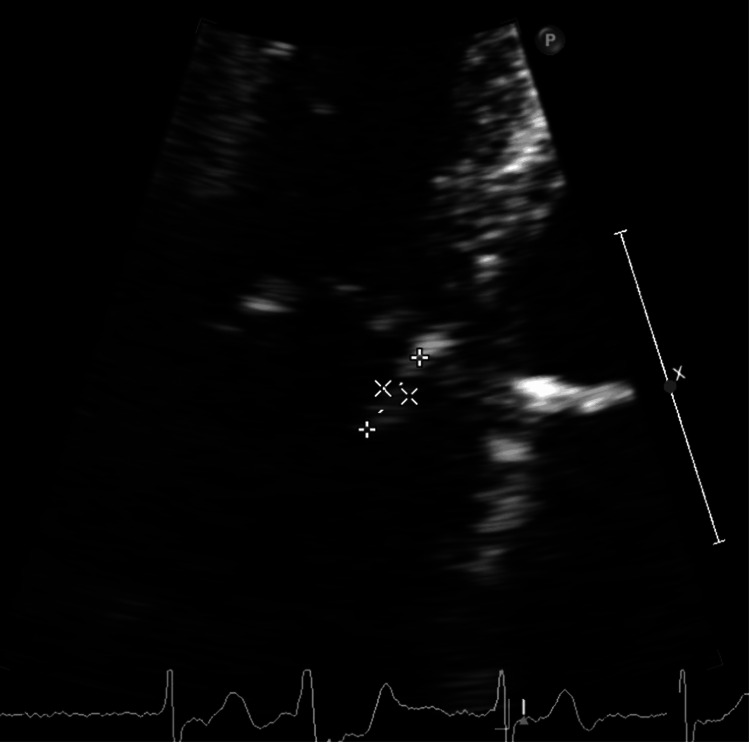
Zoomed-in view of the tricuspid valve in apical four-chambered view, redemonstrating the mobile echo density as seen in Video 2 (pointers).

A stroke code was called; however, the patient was not a candidate for reperfusion therapies due to hemorrhagic conversion and a suspicion of septic embolic stroke. The sepsis protocol was initiated: blood cultures were obtained, and the patient was started on broad-spectrum antibiotic coverage with vancomycin and cefepime. TTE showed a well-seated Edwards SAPIEN bioprosthetic valve in the aortic area (Video 1) with normal gradients but noted a mobile echo density on the septal tricuspid valve leaflet (Video 2 and Figure 2) . 

**Video 1:** TTE showing a well-seated bioprosthetic aortic valve in short axis view. TTE: Transthoracic echocardiogram

**Video 2:** TTE in apical four-chambered view (tricuspid valve zoomed in), exhibiting a 0.4 x 1 cm mobile echo density on the atrial side emerging from the base of the septal tricuspid valve leaflet. TTE: Transthoracic echocardiogram

**Figure 3:**
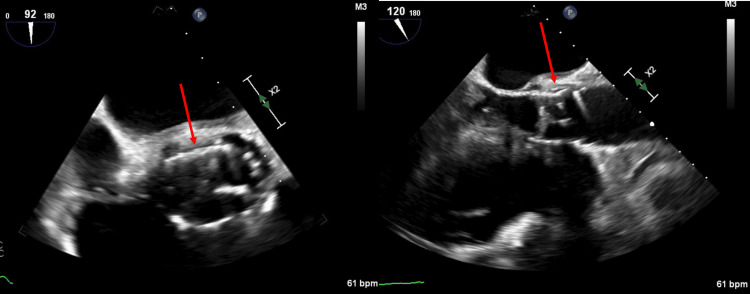
Transesophageal echocardiogram short axis aortic view (left). Transesophageal echocardiogram long axis view focusing on the aortic valve (right). TAVR: Transcatheter aortic valve replacement History of TAVR.  Note the well-seated prosthesis. Echo-free space noted (as shown by the arrow) adjacent to the mitral valve annulus in continuous with the intervalvular fibrosa. There is significant thickening and echolucency of intervalvular fibrosa suggestive of aortic root abscess

Two sets of blood cultures with polymerase chain reaction (PCR) panel identified *Haemophilus parainfluenza*, increasing the suspicion for valvular endocarditis. Transesophageal echocardiography (TEE) was pursued and revealed significant thickening and echolucency of the intervalvular fibrosa, suggesting an aortic root abscess (Figure 3). 

The patient was not deemed to be a surgical candidate given the high surgical risk. The patient's mental status improved with antibiotic therapy. Following final blood culture results and sensitivities, vancomycin was discontinued, and the patient was discharged with a peripherally inserted central catheter (PICC) to complete a six-week course of intravenous (IV) cefepime. Follow-up TTE in the clinic after completion of antibiotic therapy showed complete resolution of the aortic root abscess and the tricuspid valve vegetation.

## Discussion

IE is an uncommon but serious complication following TAVR. In spite of major advancements in the TAVR procedure, the incidence of IE after TAVR remains stable, with 0.3 to 2 per 100 person-years similar to that after surgical aortic valve replacement (SAVR) [4]. Significant risk factors for IE following TAVR encompass a previous history of IE, diabetes, chronic obstructive pulmonary disease, chronic kidney disease, pulmonary hypertension, atrial fibrillation, and receiving blood transfusions during TAVR hospitalization [5,6]. These factors are notable but not all-inclusive. Generally, the most frequent initial symptom of TAVR-related IE (TAVR-IE) is fever, observed in about 80% of cases, followed by new-onset heart failure, which appears in roughly 40% of cases [7,8]. Additionally, around 13% of patients experience systemic embolic events [9]. It is important to mention that atypical and nonspecific symptoms are more commonly seen in patients with TAVR-IE.

IE after TAVR is a specific type of prosthetic valve endocarditis (PVE), posing significant challenges due to its distinct clinical and microbiological characteristics. In high-income countries, *Staphylococci*, particularly *Staphylococcus aureus*, are the primary pathogens for both native and prosthetic valve IE [9]. However, the most common microorganisms in TAVR-IE include *Enterococci*,* S. aureus*, and coagulase-negative *Staphylococci* [8]. Although infrequent, HACEK organisms, which normally reside in the oral and upper respiratory tracts, have also been identified in TAVR-IE cases. These are implicated in about 1-3% of all IE cases due to their ability to invade blood vessels following trauma or local infection [3,10]. HACEK endocarditis often has an insidious onset, and diagnosis is typically delayed in prosthetic valves because these slow-growing bacteria frequently lead to initially negative blood cultures. While the incidence of PVE is on the rise, HACEK-related PVE is uncommon but critical to address promptly due to its potential for severe outcomes.

IE following TAVR is linked with a high incidence of serious complications, affecting up to 70% of patients in certain studies [7]. The most frequent complications reported during the initial hospital stay include acute heart failure, acute renal failure, septic shock, acute myocardial infarction, and systemic embolism [9,11]. Additionally, paravalvular issues and abscesses are relatively common, with occurrences reported between 3.6% and 19.1% [9]. Regardless of the treatment approach, IE after TAVR is associated with extremely high mortality rates. 

The Modified Duke’s criteria are less effective for diagnosing TAVR-IE than for native valve IE, mainly because of the higher frequency of negative blood cultures and ambiguous echocardiographic findings. A significant nationwide study highlighted the limited sensitivity of TTE and TEE in detecting TAVR-IE [12]. These echocardiographic studies were found to be normal or inconclusive in nearly half of the TAVR-IE cases, indicating a substantial diagnostic challenge. The American College of Cardiology and the American Heart Association have endorsed multidetector CT with a Class IIA recommendation for diagnosing TAVR PVE [13]. CT is particularly effective in identifying abscesses and pseudoaneurysms, offering diagnostic precision comparable to TEE.

Currently, there are no randomized trials comparing different antibiotic treatments or strategies for TAVR-IE, leaving treatment largely guided by general guidelines for PVE [14]. IV antibiotic therapy should be tailored based on the microorganism's profile and susceptibility tests. Initial empiric treatment should target *Staphylococci*, *Streptococci*, *Enterococci*, and Gram-negative bacteria. If an infection persists beyond one week, a reevaluation for potential alternative infection sources is necessary before considering surgery. Urgent surgical intervention for PVE may be needed for severe complications such as acute regurgitation leading to shock, heart failure due to severe obstruction, or persistent infections despite adequate therapy [15]. Despite indications for surgery in about 80% of TAVR-IE cases, surgical interventions remain exceptionally rare, with few studies reporting rates above 20% [15]. Antibiotic therapy for TAVR-IE should typically extend for at least six weeks, reflecting its classification as a subset of PVE [16]. However, the underrepresentation of TAVR patients in PVE studies means that findings from these studies might not fully apply to the TAVR-IE population. Overall, the management of TAVR-IE remains uncertain, with conservative treatment via antibiotics being the predominant strategy despite the potential benefits of surgical intervention observed in native valve endocarditis and PVE.

In this case, the patient exhibited acute mental status changes and generalized weakness due to a septic embolic stroke, driven by underlying TAVR-IE with an aortic root abscess. The presentation of sepsis after TAVR raised suspicions of PVE over other causes of embolic stroke. While TTE and TEE demonstrated aortic root abscess and tricuspid valve vegetations, indicating TAVR-IE, it is important to note that echocardiography can sometimes be less sensitive in diagnosing this condition. Nevertheless, in this instance, the echocardiograms were definitive. Blood cultures complemented with a PCR panel successfully identified *Haemophilus parainfluenza* as the causative agent, despite HACEK organisms typically being culture-negative. Although there was a clear indication for surgical intervention, the patient's multiple comorbidities placed him at high surgical risk. Consequently, the decision was made to manage the condition medically. The patient was treated with six weeks of IV antibiotics, leading to complete resolution of clinical symptoms and normalization of echocardiographic findings. 

## Conclusions

This case highlights the need for careful monitoring of TAVR patients for potential complications, such as IE. Unlike PVE or native valve endocarditis, TAVR-IE presents a unique and atypical profile. This necessitates individualized treatment plans tailored to address the specific challenges and complexities of this serious condition.

## References

[1] Coisne A, Lancellotti P, Habib G (2023). ACC/AHA and ESC/EACTS guidelines for the management of valvular heart diseases: JACC guideline comparison. J Am Coll Cardiol.

[2] Lnu K, Ansari S, Mahto S (2021). Transcatheter aortic valve replacement associated infective endocarditis case series: broadening the criteria for diagnosis is the need of the hour. BMC Cardiovasc Disord.

[3] Chambers ST, Murdoch D, Morris A (2013). HACEK infective endocarditis: characteristics and outcomes from a large, multi-national cohort. PLoS One.

[4] Ali N, Baig W, Wu J, Blackman D, Gillott R, Sandoe JA (2020). Prosthetic valve endocarditis following transcatheter aortic valve implantation. J Cardiovasc Med (Hagerstown).

[5] Bjursten H, Rasmussen M, Nozohoor S, Götberg M, Olaison L, Rück A, Ragnarsson S (2019). Infective endocarditis after transcatheter aortic valve implantation: a nationwide study. Eur Heart J.

[6] Cahill TJ, Raby J, Jewell PD (2022). Risk of infective endocarditis after surgical and transcatheter aortic valve replacement. Heart.

[7] Del Val D, Panagides V, Mestres CA, Miró JM, Rodés-Cabau J (2023). Infective endocarditis after transcatheter aortic valve replacement: JACC state-of-the-art review. J Am Coll Cardiol.

[8] Latib A, Naim C, De Bonis M (2014). TAVR-associated prosthetic valve infective endocarditis: results of a large, multicenter registry. J Am Coll Cardiol.

[9] Regueiro A, Linke A, Latib A (2016). Association between transcatheter aortic valve replacement and subsequent infective endocarditis and in- hospital death. JAMA.

[10] Khaledi M, Sameni F, Afkhami H, Hemmati J, Asareh Zadegan Dezfuli A, Sanae MJ, Validi M (2022). Infective endocarditis by HACEK: a review. J Cardiothorac Surg.

[11] Del Val D, Abdel-Wahab M, Linke A (2021). Temporal trends, characteristics, and outcomes of infective endocarditis after transcatheter aortic valve replacement. Clin Infect Dis.

[12] Stortecky S, Heg D, Tueller D (2020). Infective endocarditis after transcatheter aortic valve replacement. J Am Coll Cardiol.

[13] Nishimura RA, Otto CM, Bonow RO (2017). 2017 AHA/ACC focused update of the 2014 AHA/ACC guideline for the management of patients with valvular heart disease: a report of the American College of Cardiology/American Heart Association task force on clinical practice guidelines. Circulation.

[14] Habib G, Lancellotti P, Antunes MJ (2015). 2015 ESC guidelines for the management of infective endocarditis: the task force for the management of infective endocarditis of the European Society of Cardiology (ESC). Endorsed by: European Association for Cardio-Thoracic Surgery (EACTS), the European Association of Nuclear Medicine (EANM). Eur Heart J.

[15] Lalani T, Cabell CH, Benjamin DK (2010). Analysis of the impact of early surgery on in-hospital mortality of native valve endocarditis: use of propensity score and instrumental variable methods to adjust for treatment-selection bias. Circulation.

[16] Alexis SL, Malik AH, George I (2020). Infective endocarditis after surgical and transcatheter aortic valve replacement: a state of the art review. J Am Heart Assoc.

